# A model of healthy aging and motor inhibition in the basal ganglia

**DOI:** 10.1186/1471-2202-12-S1-P331

**Published:** 2011-07-18

**Authors:** Kyle Lyman, Joaquin Anguera, David Terman

**Affiliations:** 1 Math Department, Ohio State University, Columbus, OH 43201, USA; 2 Department of Neurology and Physiology, University of California San Francisco, San Francisco, CA, USA

## 

The basal ganglia have received a great deal of attention from modeling studies because of their putative role in normal motor function including action selection and sequencing, as well as their distubrances in Parkinson’s disease. However, their interaction is complex, and is thought to be more sophisticated than can be accounted for by simple box and plot diagrams.

In the present study, we have examined trial by trial variations in response times in the stop signal task for both young (YA) and older (OA) adults. We compare previously reported YA data [1] with data collected from 18 older adults performing the same task. Unlike YA, the OA do not show a specific pattern of response times that is modulated by stop signal success in the previous trial. Additionally, the OA did not show an effect of inter trial interval (ITI) as was observed in YA. Based on our behavioral data [1], we concluded that a task switching interpretation was the most likely explanation of the YA results.

We also compare the electrophysiological data collected during the ITI from OA and YA performing the stop signal task. In YA, the power spectral data revealed a midline frontal theta effect with more desynchronization following a stop signal trial. As in [1], we concluded that this evinced a task set switching interpretation as YA subjects went from a ‘ready to inhibit’ mode to a ‘ready to respond’ mode. In the OA data presented here, we failed to observe a similar frontal theta effect, which was expected given the lack of a behavioral result in the OA subjects.

To test hypotheses about the role of the basal ganglia in task switching in young and older adults, we have developed a conductance-based network model of the subthalamic nucleus, internal and external segments of the globus pallidus. The model incorporates cellular properties described in previous modeling studies [3] based on experimental findings. First we establish network properties which lead to patterns of beta activity that are consistent with observed phenomenon in human subjects [2]. Next, we determine which network parameters affect trial to trial variations in response times within the model. Using this approach, we examine how known changes in healthy older adult basal ganglia may affect task performance. Similarly, we discuss the implications for Parkinson’s disease.
